# Multi-Field Coupling Analysis of Resistance Spot Welding of SUS301L/Q235B Dissimilar Steel Based on Nickel Intermediate Layer

**DOI:** 10.3390/ma19112425

**Published:** 2026-06-05

**Authors:** Xiaoqi Zhang, Jinhao Li, Chengxian Yuan, Long Wang, Zhongliang Gao

**Affiliations:** 1 College of Engineering, Changchun Normal University, Changchun 130032, China; qz202310001@stu.ccsfu.edu.cn (J.L.); qz202410001@stu.ccsfu.edu.cn (C.Y.); qz202410010@stu.ccsfu.edu.cn (L.W.); 2 Quality Assurance Department, CRRC Changchun Railway Vehicles Co., Ltd., Changchun 130062, China; 013200027651@crrcgc.cc

**Keywords:** dissimilar steel linkage resistance spot welding, nickel intermediate layer multi-field coupling simulation

## Abstract

With the widespread application of stainless steel rail vehicles, the resistance spot-welding process between stainless steel and low-carbon steel has become one of the key connection processes in vehicle body manufacturing. However, due to the differences in the material physical properties of these two types of steel, problems such as center offset often occur during the welding process. This study adopts the finite element analysis method to systematically analyze the changes in the force field and the temperature field during the welding process after adding a nickel intermediate layer between the two materials, as well as its impact on the physical properties of the joint. The results of the finite element analysis and the physical experiments show that adding a nickel intermediate layer can effectively suppress the center deviation of the weld nugget, optimize the microstructure of the nugget, improve the continuity of the microhardness distribution, and thereby enhance the joint strength of the spot welding.

## 1. Introduction

Stainless steel materials have been increasingly widely used in the manufacturing of vehicle bodies due to their environmentally friendly characteristics that do not require painting [1,2,3]. At the same time, the corrosion resistance requirements for the internal parts of the vehicle body are relatively low, and low-carbon steel, with better economic properties, is often selected [4,5]. Therefore, resistance spot-welding (RSW) connection technology between low-carbon steel and stainless steel has become one of the key processes. However, when performing RSW of different metals, due to the differences in the physical properties of the materials, especially in terms of resistivity and thermal conductivity, it is easy to cause an unbalanced distribution of current and heat during the welding process, causing the weld core to lean towards the side with the lower thermal conductivity [6,7,8,9,10].

Meanwhile, under the influence of the high-temperature welding thermal cycle, brittle Fe–Cr carbides and hard brittle σ phases are prone to form at the interface of the heterogeneous materials [11,12,13]. The formation of these brittle phases significantly reduces the toughness of the joint and has an adverse effect on fatigue life [14,15,16,17,18]. Therefore, using an interlayer to regulate the interface reaction and the thermodynamic behavior is a well-known method to improve welding performance in heterogeneous metal connections [19,20,21,22,23]. The commonly used intermediate metal layers mainly include pure metals, alloys, and composite materials [24,25,26]. The core advantage of pure metal intermediate layers lies in their single composition and structure, which brings more predictable and stable metallurgical diffusion behavior and simpler interface reaction control [27,28,29,30]. Pure metal intermediate layers include nickel and copper [31,32,33], among which nickel can form continuous solid solutions with chromium in stainless steel and iron in low-carbon steel, effectively inhibiting the formation of brittle intermetallic compounds; at the same time, its thermal expansion coefficient is between the two, which can alleviate the welding thermal stress and ensure the metallurgical compatibility and the mechanical continuity of the joint. Currently, using a nickel-based material as the intermediate layer and studying the changes in the mechanical properties of the joint is a mainstream research direction, but most existing studies are concentrated on process experiments and lack a systematic quantitative analysis of the electro–thermal–mechanical–metallurgical coupling process. The thermal–mechanical coupling simulation based on the finite element method has become the key tool for analyzing the physical essence of heterogeneous metal spot welding [34,35,36]. It can systematically reproduce the transient temperature field and the stress–strain responses. The research shows that such numerical simulations can not only deeply reproduce the joint-forming behavior but can also achieve the digital optimization of the process conditions through parameter sensitivity analysis. They demonstrate significant engineering value in replacing expensive physical experiments and guiding the pre-judgment of the joint quality in specific service environments.

This paper aims to establish a multi-field coupling numerical model for the RSW of SUS301L/Q235B heterogeneous steel, focusing on studying the regulatory mechanism of the nickel intermediate layer on the pressure distribution, the current distribution, the thermal cycle, and the nucleation morphology during welding, fundamentally explaining how the nickel intermediate layer affects the quality of the RSW joint between stainless steel and low-carbon steel, and providing a theoretical basis for engineering applications.

## 2. Experimental Materials, Equipment and Techniques

Based on the actual working conditions at the production line, the SUS301L stainless steel plates (China Jilin Province) and the Q235B low-carbon steel plates (China Jilin Province) with a thickness of 2 mm each were adopted. The thickness of the nickel interlayer ranged from 0.3 mm to 1 mm. The electrode types were as follows: RWMA class 2, type C, and 8 mm tip diameter (5RW taper) (China Jilin Province). The chemical compositions of the plates and the interlayer are shown in Table 1, and the mechanical properties are shown in Table 2.

**Figure 1:**
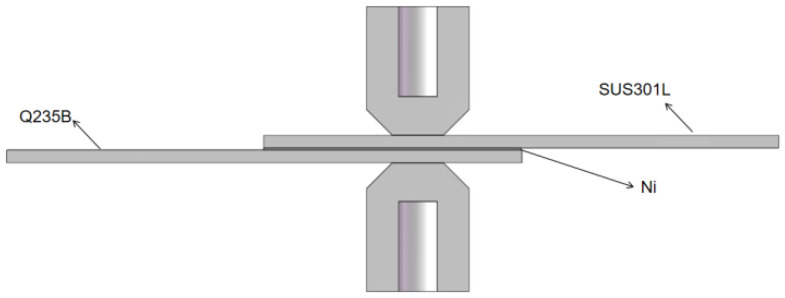
Schematic diagram of the welding test.

The welding test was conducted to form lap joints, as shown in Figure 1. The SUS301L worksheet was in contact with the upper electrode, and the Q235B worksheet was in contact with the lower electrode. The nickel interlayer was located between the steel worksheets. The thickness of the steel worksheets was 2.0 mm, which were cut into standard specimens of 138 mm × 60 mm. The size of the nickel layer was 45 mm × 60 mm, and its thickness was determined based on the finite element analysis results. It was placed at the center of the lap joint area. The welding specimens were cut perpendicularly to prepare the cross-sectional metallographic samples that included the nugget, the heat-affected zone, and the base metal zone. These samples were observed under an optical microscope to examine the morphology of the weld core, the microstructure characteristics, and the interface conditions.

**Figure 2:**
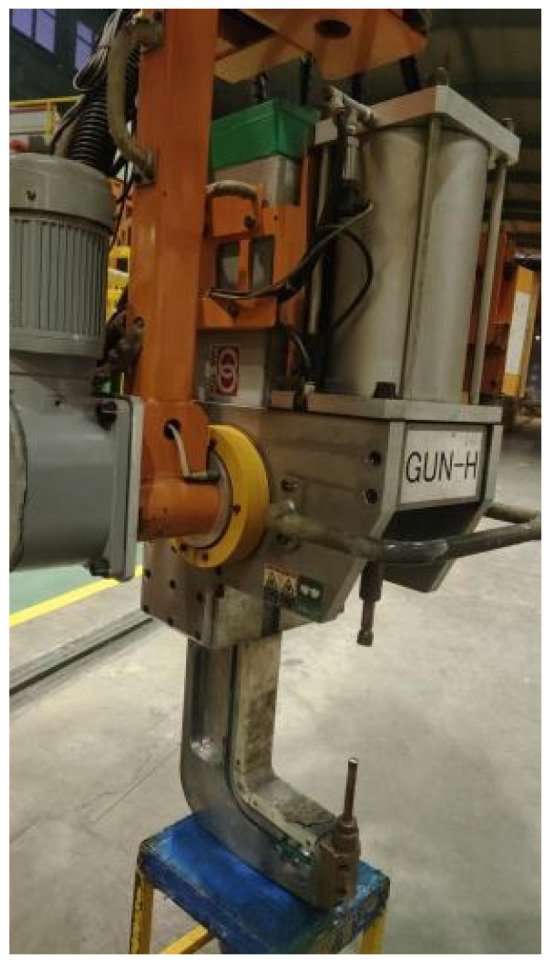
Automatic RSW equipment.

The welding equipment adopted the GUN-H automatic medium-frequency inverter direct current RSW machine (Juntengfa Industrial (Shanghai) Co., Ltd., Shanghai, China), as shown in Figure 2. This welding machine converts a three-phase alternating current through rectification, inversion, voltage reduction and full-wave rectification to output a stable direct current, which can improve the stability of heat accumulation, suppress spatter, and increase energy utilization by more than 50%. The welding parameters are shown in Table 3.

## 3. Finite Element Model

### 3.1. Finite Element Modeling

**Figure 3:**
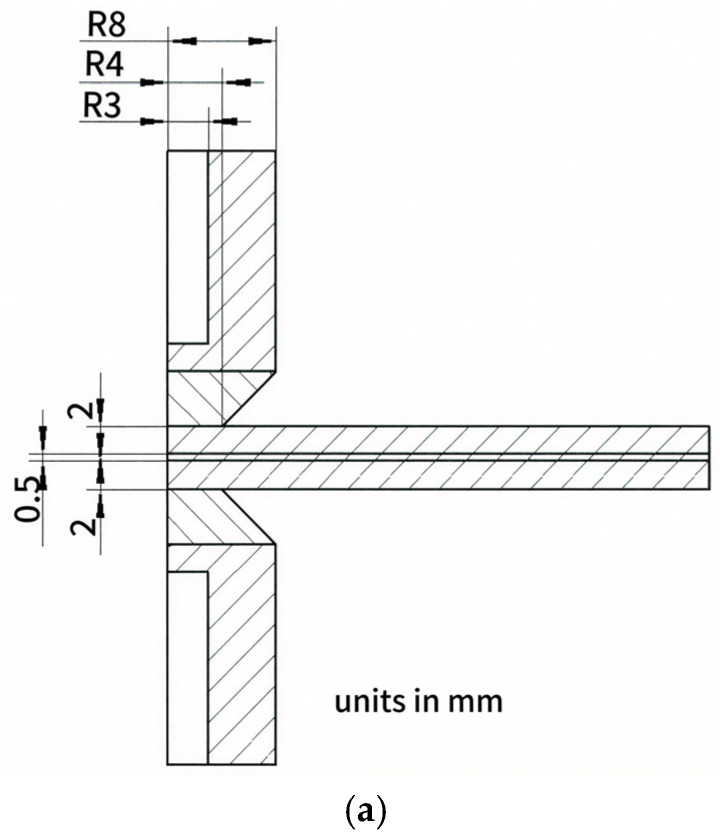

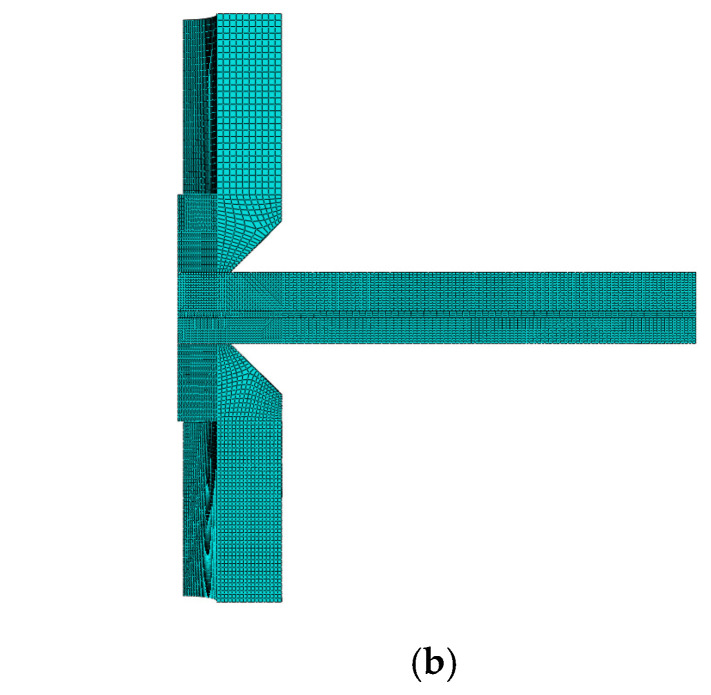
Schematic diagram of the three-dimensional model. (**a**) The dimensions; (**b**) The mesh division.

Due to the strong nonlinearity and multi-physics field coupling characteristics of the RSW process, to thoroughly study the effect of the nickel intermediate layer on the joining mechanism of SUS301L stainless steel and Q235B low-carbon steel, two dissimilar materials, this study comprehensively considers the thermal factors and the coupling effects of the electrical and mechanical fields. The size and the mesh division of the finite element analysis model are shown in Figure 3.

### 3.2. Finite Element Mesh Partition

In the finite element analysis of RSW, using the model of dissimilar steel with a nickel intermediate layer, a layer-by-layer division and a local mesh densification method was adopted. In the electrode–sheet contact area and the interface between the plates, a fine mesh of 0.2 mm was used to accurately capture the contact behavior and the current and temperature gradients. For the non-core areas, a transition mesh of 0.3–0.5 mm was adopted to improve the computational efficiency. At the interface, a consistent mesh topology was adopted, and the Q3D8R fully integrated thermal–electric coupling element was selected to ensure the accuracy and convergence of the multi-physics field coupling analysis, providing a reliable mesh foundation for the simulation.

### 3.3. Boundary Conditions and Load Settings

The boundary conditions were set according to the axisymmetric characteristics. A uniform electrode pressure load was applied to the upper electrode, and the lower electrode was completely fixed. The current path was uniformly input on the top of the upper electrode, and the zero potential point was set at the bottom of the lower electrode. The thermal boundary condition adopted the equivalent convective heat transfer model, with the environmental temperature kept constant at 20 °C. The comprehensive heat transfer coefficient of the electrode cooling channel was 3800 W/(m^2^ °C). The model ensures calculation convergence through the following idealization processing [37,38,39,40]:

(1) Ignoring material self-weight and radial displacement effects during welding processes.
(2) Using instantaneous linear strengthening models to characterize material stress–strain relationships.
(3) Maintaining symmetry in the entire model, load distribution, and boundary conditions.

In elastoplastic finite element theory, simulating nonlinear material deformation during RSW requires three fundamental principles: the yield criterion for identifying plastic deformation initiation, the strengthening criterion describing the post-yield hardening behavior, and the flow law determining the direction of the plastic strain increments. In thermal conduction analysis, temperature serves as the core physical quantity, expressed as a continuous function of spatial coordinates and time. Based on Fourier heat conduction laws and energy conservation principles, the governing equations for non-steady heat transfer processes can be derived, which govern the evolution of transient temperature fields [41]:

(1) $$\frac{\partial }{\partial x}\left(K_{x}\frac{\partial T}{\partial x}\right)+\frac{\partial }{\partial y}\left(K_{y}\frac{\partial T}{\partial y}\right)+\frac{\partial }{\partial z}\left(K_{z}\frac{\partial T}{\partial z}\right)+\rho Q\left(x,y,z,t\right)=\rho c_{T}\frac{\partial T}{\partial t}$$

In the above equation, ρ denotes the material density;$\;c_{T}\;$represents the specific heat capacity;$\;K_{x}{,K}_{y}{,andK}_{z}\;$ denote the thermal conductivity coefficients in the *x*, *y*, and *z* directions, respectively; while $\;Q\left(x,y,z,t\right)\;$ indicates the internal heat source intensities within the component.

The fundamental physical quantity in the electric potential field is the electric potential function, which is mathematically represented as a continuous variable in space and time. For a RSW model with an axisymmetric geometry, the classical electromagnetic theory framework can describe the axisymmetric electric field distribution through the nonlinear Laplace equation. The solution domain includes the workpiece body, the molten pool, the adjacent solid region, and the electrode contact interface. Considering the situation where the material conductivity changes nonlinearly with the temperature, the governing equation of the electric potential field can be expressed as follows [42]:

(2) $$\frac{1}{r}\frac{\partial }{\partial r}\left(r\partial \frac{\partial \phi }{\partial r}\right)+\frac{\partial }{\partial z}\left(\sigma \frac{\partial \phi }{\partial z}\right)=0$$

In the formula, r denotes the radial coordinate, z the axial coordinate, ϕ  the electric potential, and σ the material conductivity.

### 3.4. Material Attribute

Copper electrodes, SUS301L stainless steel and Q235B low-carbon steel are regarded as isotropic homogeneous materials, and the Von Mises yield criterion [43] is adopted to describe the elastoplastic behavior of the materials. Since the high-temperature mechanical properties of the materials involved in the model, including Young’s modulus, Poisson’s ratio, density, thermal conductivity, electrical conductivity and specific heat, are difficult to obtain through experiments, they are calculated using the JMatpro7.0 software. In the simulation, due to the introduction of the nickel layer, the model has four contact pairs, namely the electrode–low-carbon steel contact pair, the low-carbon steel–nickel intermediate layer contact pair, the stainless steel–nickel intermediate layer contact pair, and the electrode–stainless steel contact pair. Since the properties of the materials at different temperatures can affect the accuracy of the simulation results, when both current and pressure act simultaneously, the factor that the numerical values of the thermal and electrical properties of the materials will change with temperature needs to be considered [44]. The contact resistance between different metal materials is calculated using the Wanheim–Bay Model Formula (1):

(3) $$\rho_{\mathrm{contact}}\;=\;3\frac{\sigma_{s_{-soft}}}{\sigma_{n}}\frac{\rho_{1}+\rho_{2}}{2}\;+\;\gamma \rho_{\mathrm{contaminants}}$$

In the Formula, the flow stress of the material is represented, and the contact pressure is represented. They represent the resistivity of the two different materials, which are the correction factors and the influence of additives in the material itself. When the contact distance is greater than 0.01, the contact properties is set as the non-contact properties of the contact interface.

## 4. Finite Element Analysis Results and Discussion

### 4.1. Preloading Stage

#### 4.1.1. Stress Field and Contact Pressure Analysis

**Figure 4:**
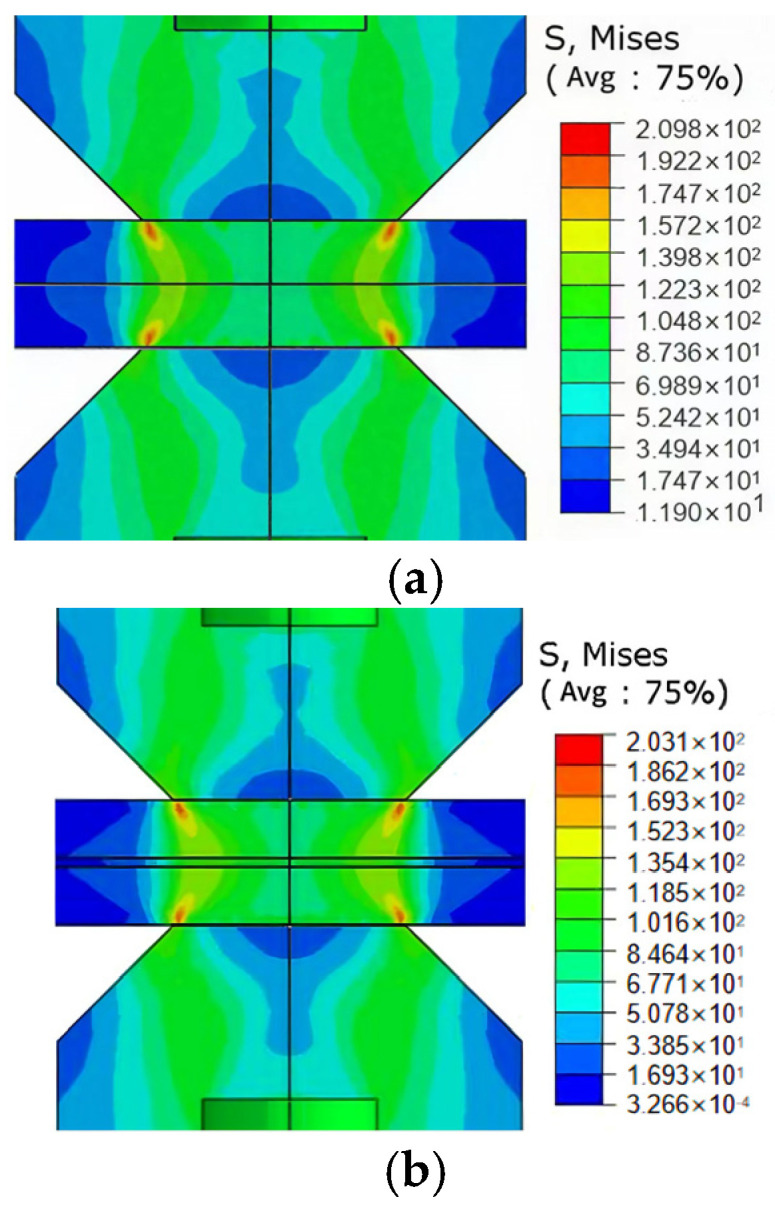
Distribution of equivalent stress in the preloading stage. (**a**) Equivalent stress distribution without nickel intermediate layer added; (**b**) Equivalent stress distribution with nickel intermediate layer added.

Under an electrode pressure of 8 kN, Figure 4 shows the simulation analysis results of the equivalent stress during the preloading stage. From Figure 4a, when no nickel interlayer was added, the peak equivalent stress reached 209.7 MPa, concentrated in the edge area where the electrode end face contacts the base material. This is in line with the typical distribution characteristics of contact mechanics during RSW. Due to the limited plastic deformation of the plate under electrode pressure, the overall stress distribution within the sheet is relatively uniform, with relatively small numerical fluctuations. Figure 4b shows the stress distribution after adding nickel as an intermediate layer. At this time, the maximum equivalent stress slightly decreased to 203.1 MPa, and it was still located at the contact edge between the electrode and the steel plate. However, the overall stress distribution pattern shows a significant optimization: the stress peak decreased, the stress gradients became more gradual, the differences between the adjacent stress intervals reduced, and the continuity and uniformity of the distribution significantly increased. This indicates that the addition of the nickel layer effectively alleviated the interface stress concentration caused by the stiffness difference between Q235 and SUS301L, improved the transfer behavior of the electrode pressure at the contact interface, provided a more uniform stress basis for the subsequent welding process, and was conducive to enhancing the structural integrity and service reliability of the joint.

**Figure 5:**
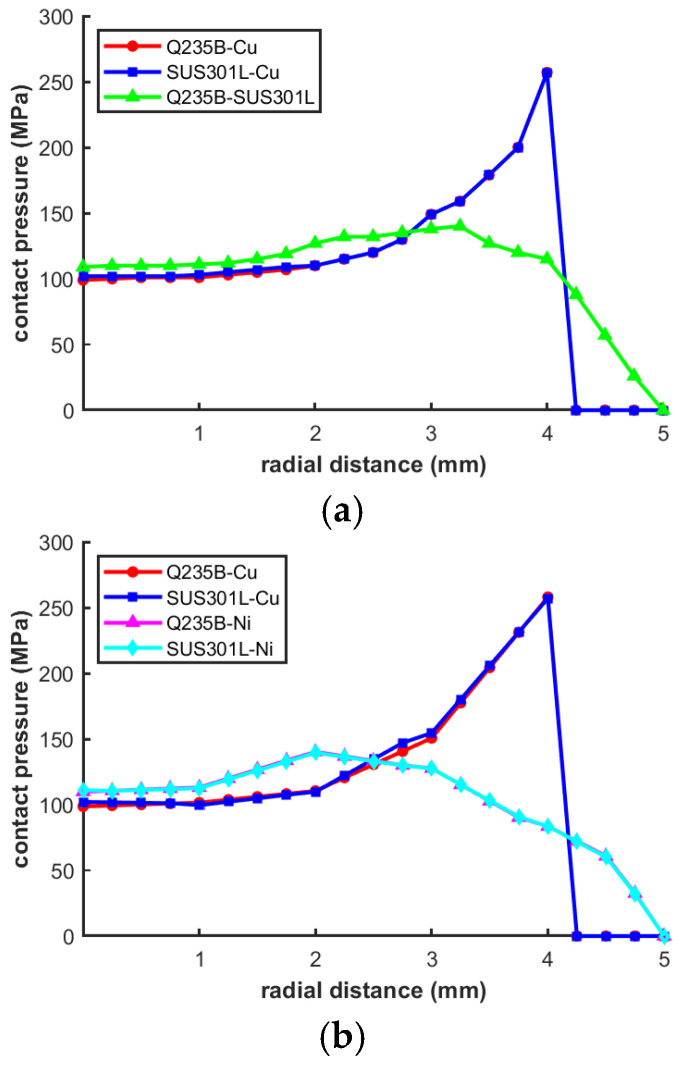
Distribution of contact pressure during the preloading process. (**a**) Pressure distribution at the contact surface without nickel intermediate layer added; (**b**) Pressure distribution at the contact surface with nickel intermediate layer added.

Figure 5 shows the distribution characteristics of the contact pressure at the electrode–workpiece and the workpiece–workpiece interfaces during the preloading process. Figure 5a shows the contact pressure distribution without adding a nickel interlayer. The results indicate that the contact stress distribution curve of the Cu-301L side is highly consistent with the Cu-Q235B side. However, the overall contact pressure on the Cu-Q235B side is slightly higher than that on the Cu-301L side. A significant stress concentration was observed at the edge area of the contact surface between the electrode and the workpiece. Within the radial range of 0–3 mm, the contact pressure distribution was relatively smooth, indicating that a stable contact state had been achieved between the electrode and the workpiece. Within a radial range of 3–4 mm, the contact pressure significantly increased and reached its peak at 4 mm. The maximum stress at the interface between the workpieces occurred at a radial position of 3.3 mm. Within the range of 0–3.3 mm, the contact stress between the workpieces also showed a steady upward trend, but the increase was slightly greater than that at the electrode–workpiece interface. As the radial distance further increased, the contact pressure gradually decreased and became zero at 5 mm. This contact pressure distribution characteristic clarifies the range of the initial conductive area in RSW, providing a theoretical basis for the key boundary conditions of the subsequent thermoelectric–force multi-physical field coupling simulations.

Figure 5b shows the distribution of the contact surface pressure after adding the nickel interlayer. The results indicate that the overall pressure peak shows almost no significant change compared to when no nickel layer was added. The peak at the contact surface between the electrode and the workpiece appeared at approximately 4 mm, and the variation in the pressure peak was more gradual; the peak of the contact pressure between workpieces appeared at about 2 mm, and the contact pressure decreased more gently and smoothly within the 2–5 mm range. Although adding a nickel layer does not change the peak of the contact pressure, it has a fine-tuning effect on the stress transfer characteristics of each contact interface. This adjustment helps optimize the distribution of the contact resistance and provides more detailed theoretical guidance for the precise control of RSW processes.

#### 4.1.2. Influence of Electrode Pressure on the Preloading Process

**Figure 6:**
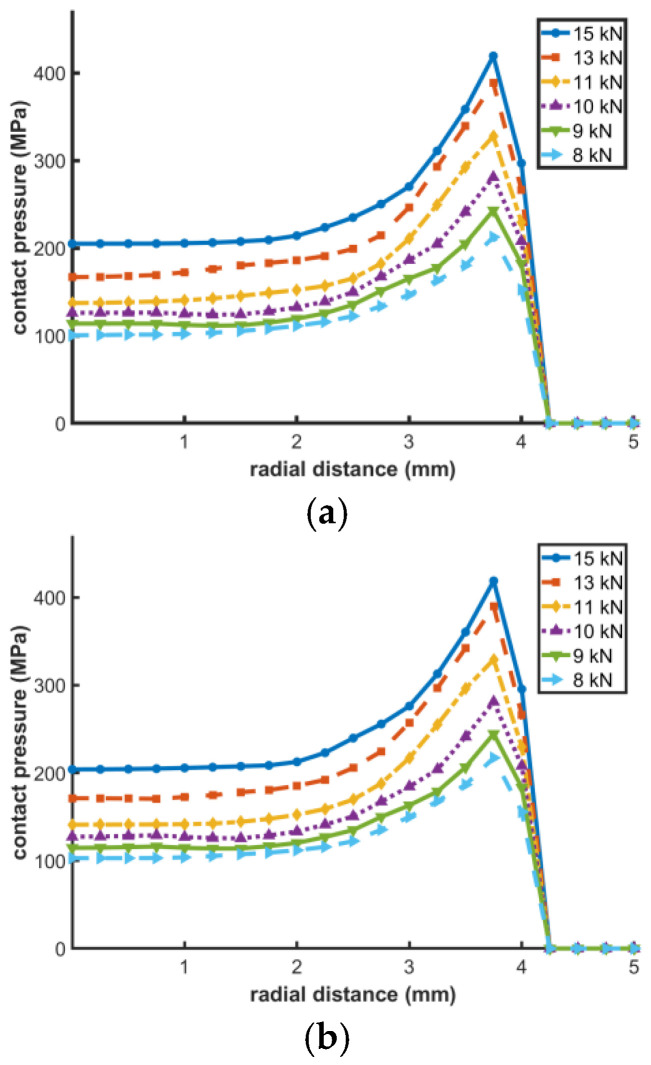
Contact pressure distribution between workpiece and electrode under different electrode pressures. (**a**) Contact pressure distribution between Q235B and the electrode; (**b**) Contact pressure distribution between SUS301L and the electrode.

**Figure 7:**
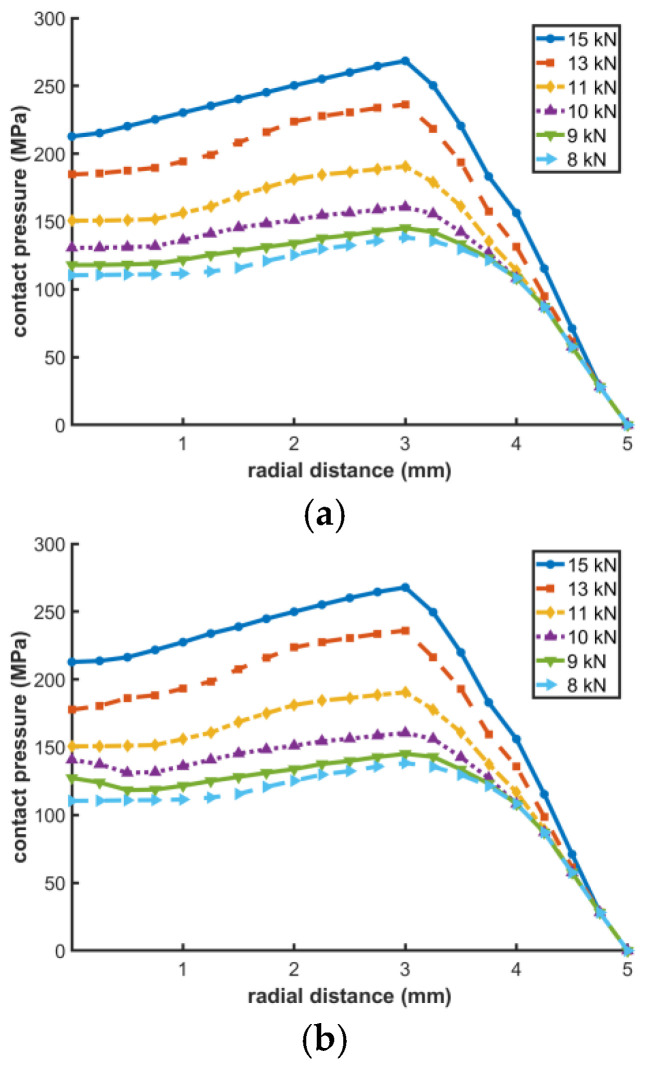
Workpiece–workpiece contact pressure distribution under different electrode pressures. (**a**) Contact pressure distribution between Q235B and the Ni; (**b**) Contact pressure distribution between SUS301L and the Ni.

To study the influence of the nickel interlayer on the contact pressure under different electrode pressures, the electrode pressure was set to 8 kN, 9 kN, 10 kN, 11 kN, 13 kN and 15 kN. The contact pressure distributions between the workpieces and between the electrode and the workpiece were obtained, and the results are shown in Figure 6 and Figure 7.

Figure 6a presents the contact pressure distribution between the electrode and Q235B, while Figure 6b shows that between the electrode and SUS301L. As illustrated in Figure 6, as the electrode pressure increases, the trend of contact pressure variation between workpieces remains unchanged, with an overall increase in the numerical values.

Figure 7 shows the pressure distribution on the Q235B–Ni and Ni–301L contact surfaces under different electrode pressures. As the electrode pressure increases, the contact pressure between the workpieces generally rises, but the location of the maximum value does not change with the increase in electrode pressure and consistently occurs at a radius of 3.30 mm. This indicates that the maximum contact pressure between the workpieces is influenced by the electrode size and pressure, while the contact radius is entirely determined by the radius of the electrode size.

#### 4.1.3. Influence of Nickel Layer Thickness on the Preloading Process

**Figure 8:**
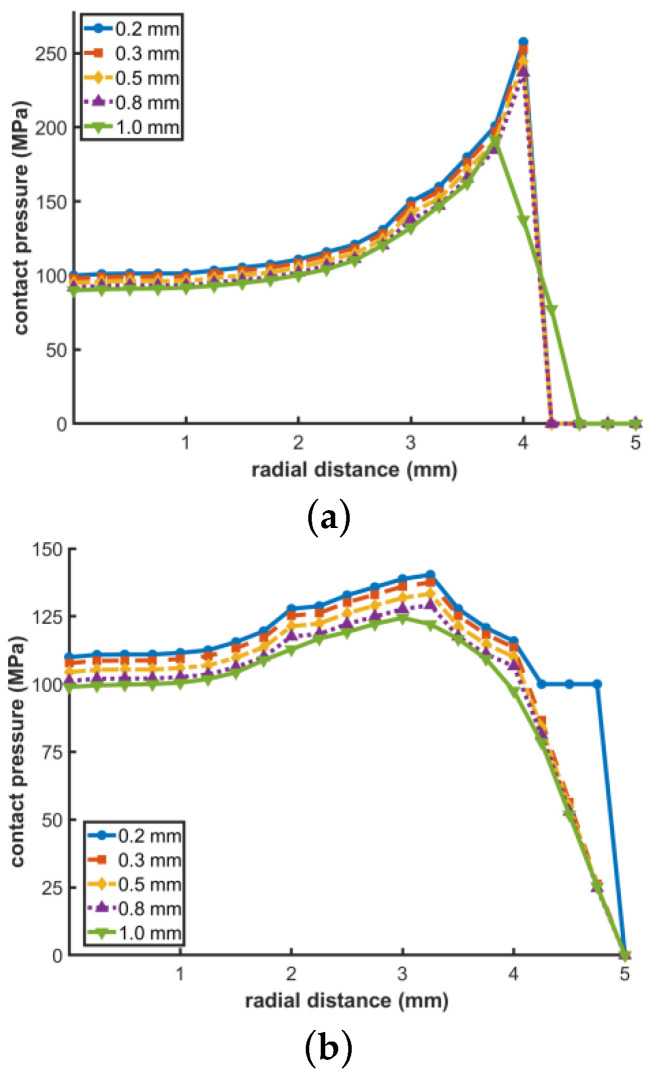
Contact pressure distribution under different nickel layer thicknesses. (**a**) Workpiece–electrode contact pressure; (**b**) Workpiece–workpiece contact pressure.

To study the influence of the nickel layer thickness on the contact pressure, the electrode pressure was set to 8 KN, and the thickness of the nickel layer was set to 0.2 mm, 0.3 mm, 0.5 mm, 0.8 mm and 1.0 mm. Since the contact stress distribution on the SUS301L side is almost the same as that on the Q235B side, the contact stress distribution on the Q235B side was adopted to represent both. The changes in contact pressure are shown in Figure 8.

From Figure 8a, it can be observed that as the thickness of the nickel layer increases from 0.2 mm to 1.0 mm, the peak contact pressure decreases. At a thickness of 0.2 mm, the peak pressure is the highest, reaching 257.6 MPa. However, it drops to approximately 231.8 MPa at 1.0 mm. For every 0.1 mm increase in thickness, the peak pressure decreases by about 3.2–3.5 MPa. Under a thin nickel layer, the pressure distribution is more concentrated, and the pressure gradient is larger. The peak value is reached when the radial distance is approximately 3.5 mm. This helps achieve a concentrated heat input, but it may lead to a concentration of local stress.

The contact pressure distribution at the Q235–Ni interface under different nickel layer thicknesses is shown in Figure 8b. As the nickel layer thickness increases from 0.2 mm to 1.0 mm, the peak contact pressure decreases from approximately 142.3 MPa to about 126.3 MPa, with a reduction of 3–4 MPa for every 0.1 mm increase in thickness. This is attributed to the stress-buffering effect of the nickel layer acting as a soft intermediate layer. A thin nickel layer can lead to a concentrated pressure distribution and a steep pressure gradient, which is conducive to forming a deep penetration nugget but prone to splashing and electrode wear. A medium-thickness nickel layer achieves a good balance between reducing the pressure concentration and ensuring a uniform pressure distribution, suitable for conventional welding and mass production. A thick nickel layer has the smoothest pressure distribution and the smallest pressure gradient, which can effectively alleviate stress concentration, extend the electrode life, and prevent the oxidation of the base material surface. This is particularly advantageous for thin plate welding and applications requiring a high surface quality.

### 4.2. Electrode Energizing Phase

#### 4.2.1. Electric and Temperature Field Simulations with and Without a Nickel Layer

**Figure 9:**
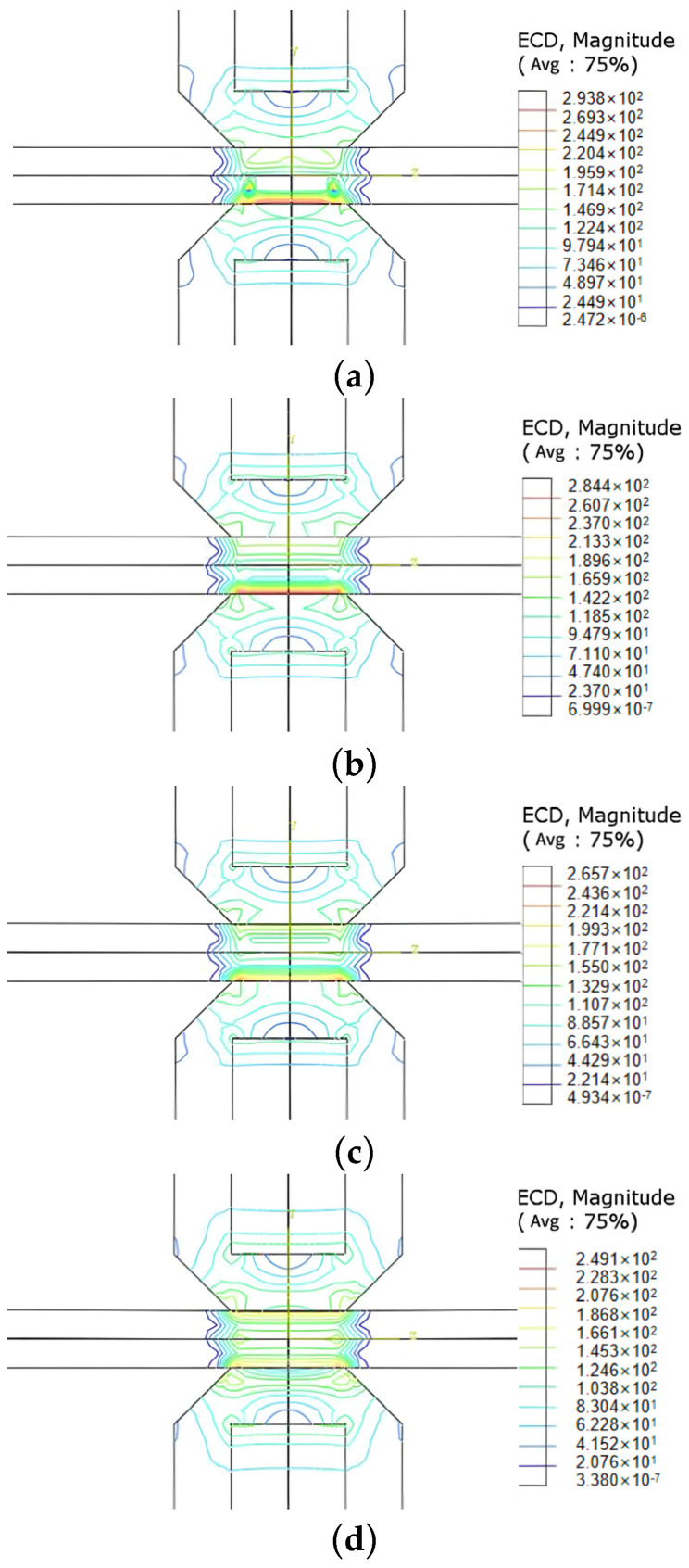
Current density distribution at different electric application times without a nickel layer. (**a**) The electric application time is 0.1 s; (**b**) The electric application time is 0.2 s; (**c**) The electric application time is 0.3 s; (**d**) The electric application time is 0.4 s.

**Figure 10:**
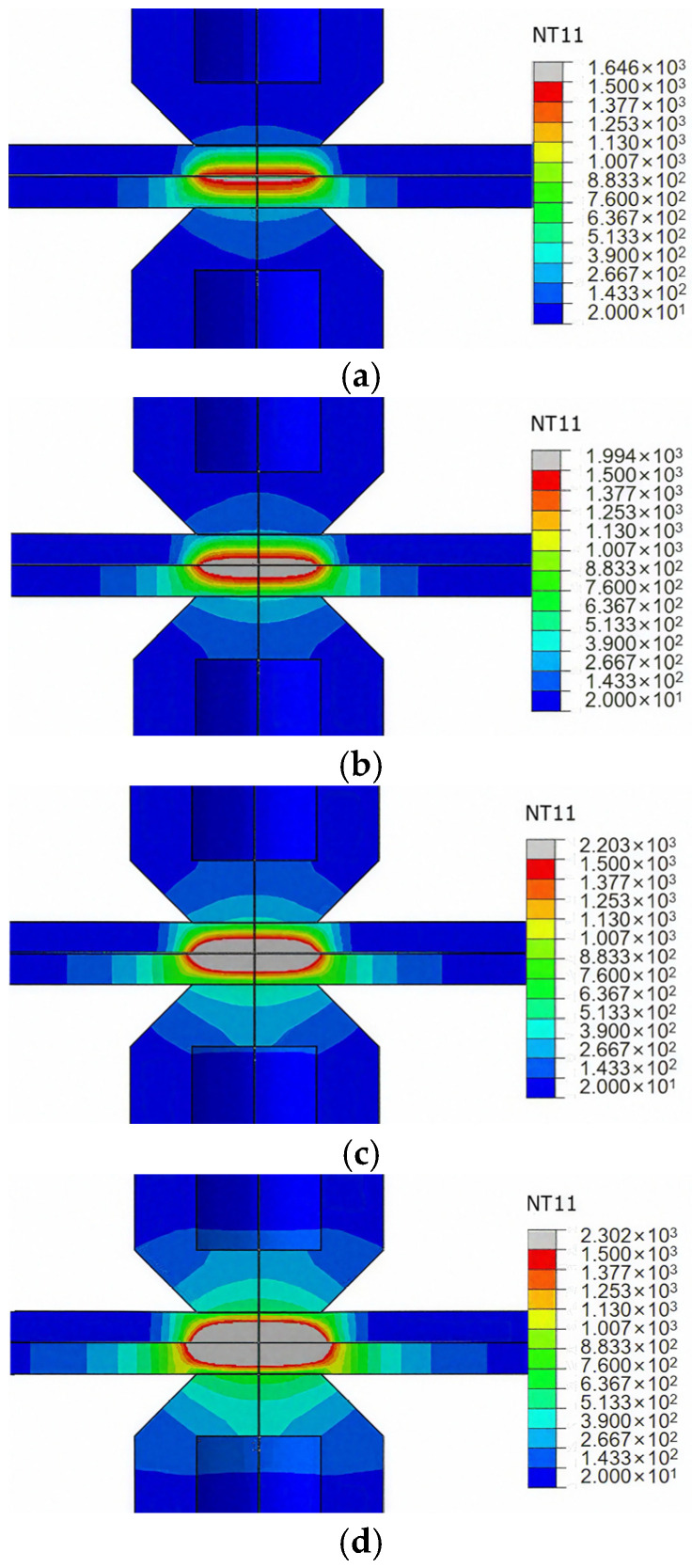
Temperature field simulation results at different electric application times without a nickel interlayer. (**a**) The electric application time is 0.1 s; (**b**) The electric application time is 0.2 s; (**c**) The electric application time is 0.3 s; (**d**) The electric application time is 0.4 s.

After the preloading process is completed, while keeping the electrode pressure unchanged, an electric load is applied to conduct the calculation of the thermal–electro-structural coupling model, and the changes in the current density and temperature field within the workpiece during the energization process are solved. When no nickel interlayer was added, the transient current density and the temperature field distribution within the workpiece during each stage of power application are shown in Figure 9 and Figure 10.

As shown in Figure 9, during the RSW process, the current density distribution changes significantly over time. In the initial period of 0.1 s, due to the difference in the contact resistance and the higher resistivity of stainless steel, the current is concentrated at the interface between the electrode and the low-carbon steel. As the time progresses to 0.2 s, the contact point softens due to heating, the contact resistance decreases, and the overall temperature rises, causing a change in material resistance. The current peak decreases and shifts towards the side with better conductivity, the low-carbon steel. After 0.3 s, the weld nucleus begins to form, and the conductivity of the liquid metal area increases. The current distribution becomes more uniform, and the peak continues to fall. At 0.4 s, the weld nucleus stabilizes, and the temperature of the workpiece reaches its peak. The overall current density drops to a lower level and becomes more evenly distributed, with only the edge of the weld nucleus showing a slight difference due to the temperature gradient.

To clearly display the nugget formation area, the upper limit of the temperature field display is set to the material melting point temperature, where the melting point of SUS301L stainless steel is approximately 1450 °C, that of Q235B low-carbon steel is approximately 1500 °C, and that of the nickel interlayer is approximately 1500 °C.

Figure 10 shows the characteristics of the temperature field distribution without a nickel interlayer. The simulation results indicate that the high-temperature region is significantly shifted towards the SUS301L stainless steel side, with the maximum temperature appearing inside the stainless steel plate. This is consistent with the current density distribution trend shown in Figure 9. This phenomenon arises due to the differences in the physical properties of the materials: the electrical resistivity of SUS301L stainless steel is significantly higher than that of Q235B low-carbon steel. According to Joule’s law of heating, under the same current density conditions, the heat generated on the stainless steel side is approximately six times that on the low-carbon steel side. Simultaneously, the lower thermal conductivity of stainless steel restricts heat diffusion to the surrounding areas, leading to a larger temperature gradient difference and further intensifying local heat accumulation. Due to the higher electrical resistivity, lower thermal conductivity, and relatively lower melting point of SUS301L stainless steel, its nugget diameter and penetration depth are both greater than those on the Q235B low-carbon steel side. This asymmetric temperature distribution results in a significant shift of the nugget towards the stainless steel side, forming an asymmetric nugget morphology characteristic, which will adversely affect the mechanical properties and service reliability of the welded joint. The asymmetric distribution of the nugget can cause stress concentration, reduce the fatigue life of the joint, and may become a potential location for crack initiation.

**Figure 11:**
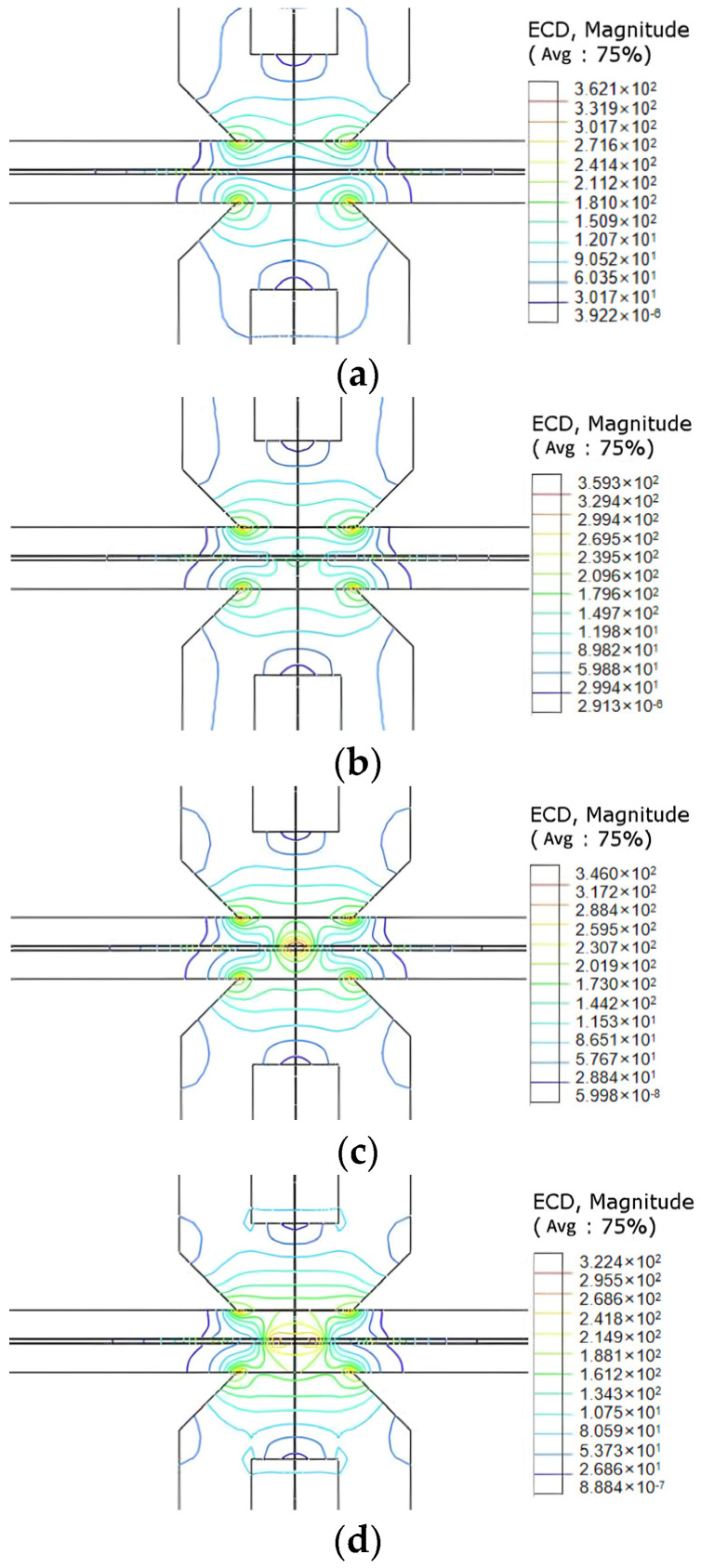
Current density distribution at different electric application times with a nickel layer. (**a**) The electric application time is 0.1 s. (**b**) The electric application time is 0.2 s. (**c**) The electric application time is 0.3 s. (**d**) The electric application time is 0.4 s.

**Figure 12:**
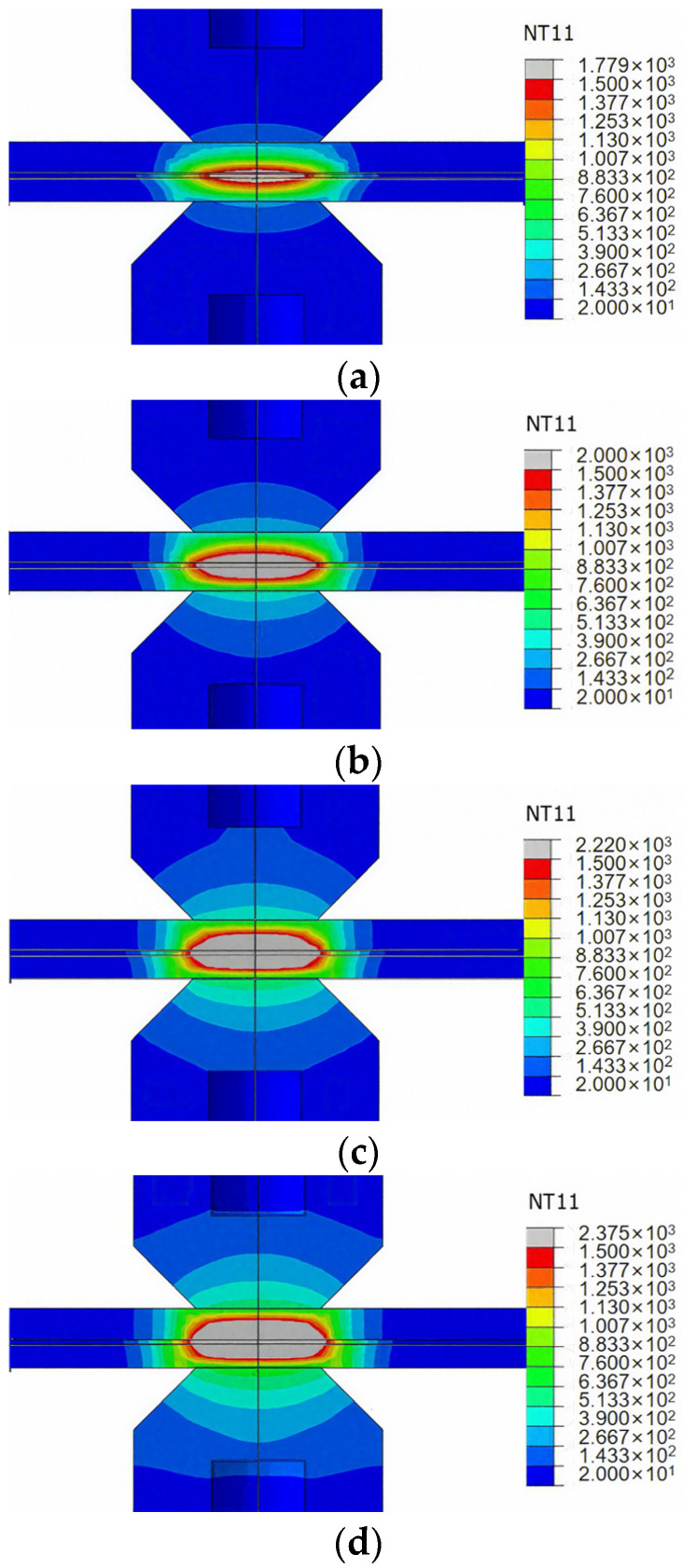
Temperature field simulation results at different electric application times with a nickel interlayer. (**a**) The electric application time is 0.1 s. (**b**) The electric application time is 0.2 s. (**c**) The electric application time is 0.3 s. (**d**) The electric application time is 0.4 s.

When a nickel interlayer is introduced between the steel plates, significant changes occur in both the current density and the temperature field distribution, as shown in Figure 11 and Figure 12.

As shown in Figure 11, during the resistance spot-welding process with the addition of a nickel interlayer, within the initial 0.1 s, the current is highly concentrated at the interface between the electrode and the adjacent base material due to the dominant role of contact resistance. As the power-on time extends to 0.2 s, the contact resistance rapidly decreases due to thermal softening. The intermediate resistivity of the nickel layer enables a more uniform heat conduction, causing the current peak to decrease and mainly to be distributed in the nickel layer and the contact area between the electrode. Around 0.3 s, the interface achieves metallurgical bonding, and the contact resistance basically disappears. The current further uniformly penetrates the nickel layer and the heat-affected zone. Eventually, a fusion zone is formed at approximately 0.4 s, and the overall conductivity becomes consistent, with the current density distributed uniformly and stabilizing at a relatively low level. Only at the fusion edge, due to the temperature gradient, are there slight differences.

Introducing a nickel interlayer alters the current transmission path. The resistivity of nickel lies between that of stainless steel and low-carbon steel, allowing it to act as a resistive transition buffer that smooths the resistance jump across the interface. This forces current redistribution as it passes through the stainless steel–nickel–low-carbon steel sandwich structure, effectively dispersing the current concentration that originally occurred at a single interface. Furthermore, the nickel layer modifies the overall resistance network of the contact, reducing the non-uniformity of the contact resistance caused by the differences in material hardness and surface conditions, thereby achieving homogenization of the interfacial conductivity. The simulation results show that the optimized two-dimensional distribution of the current density field exhibits significantly improved symmetry, substantially moderated radial gradients, and straighter current line trajectories.

Figure 12 presents the temperature field distribution and nugget formation characteristics with a nickel interlayer. The simulation results indicate that, compared with the finite element analysis results in Figure 10, the introduction of the nickel interlayer significantly improves the welding thermal process. The addition of a nickel interlayer transforms the Joule heat source from a highly asymmetric concentrated mode into a distribution pattern that is more uniform along the thickness direction and gentler along the radial direction. This drives the formation of a more symmetric temperature field and isotherms with respect to the interface. It promotes balanced nugget nucleation and growth on both sides of the interface, enhancing the geometric symmetry of the joint and the uniformity of mechanical load bearing. Simultaneously, the smoothed heat input reduces thermal shock and residual stress while suppressing expulsion. The nickel layer alters the contact resistance characteristics of the original interface and optimizes the heat distribution during the initial stage of nugget formation. This significantly reduces the deviation of the high-temperature zone in the temperature field and relocates the highest temperature area to the central axis on the Q235B side.

Compared with the condition without a nickel layer, the nugget obtained after adding the nickel interlayer exhibits a fuller and more continuous geometric shape. The nugget diameter increased by approximately 20%, the nugget height increased by about 25%, and the symmetry was significantly improved. This optimized nugget morphology helps to enhance the load-bearing capacity and fatigue performance of the joint, providing an effective process solution for controlling the welding quality of dissimilar steels.

#### 4.2.2. Influence of Nickel Layer Thickness on the Electric and Temperature Field

**Figure 13:**
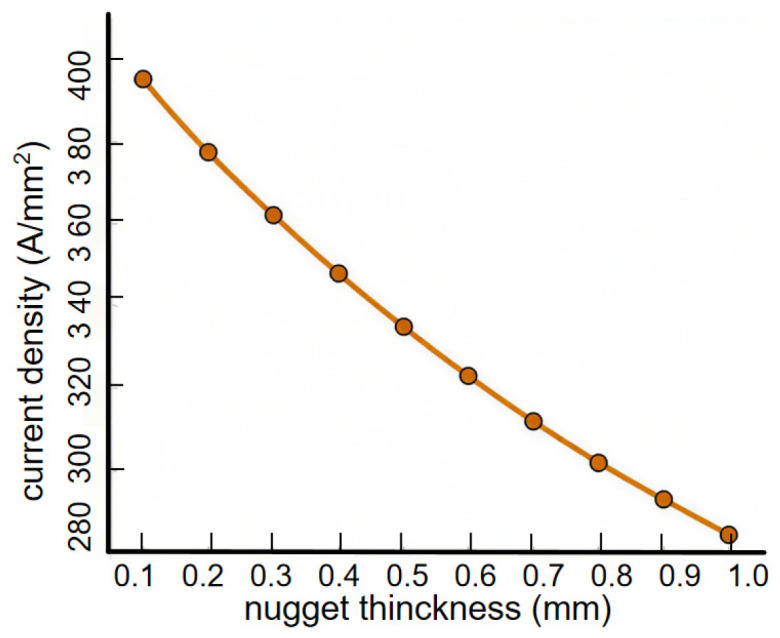
Effect of nickel layer thickness on current density.

**Figure 14:**
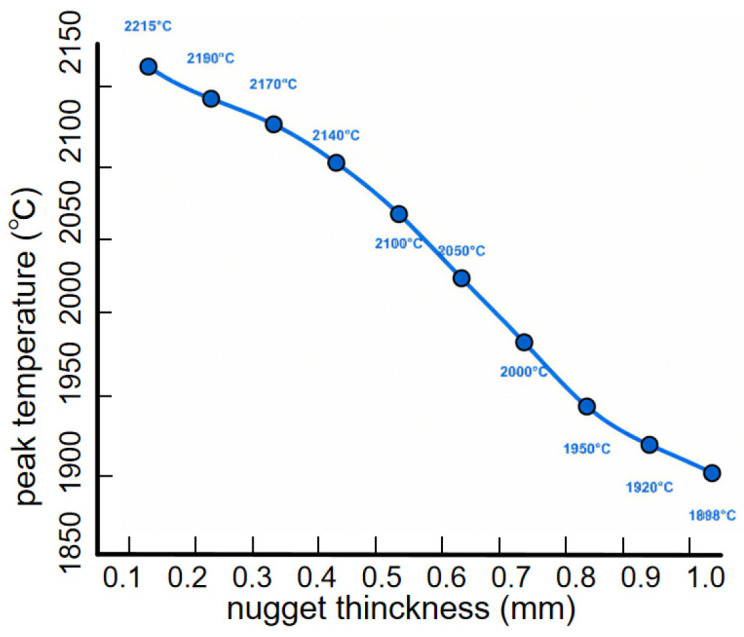
Effect of nickel layer thickness on peak welding temperature.

When the thickness of the nickel layer changes, the current density and the peak temperature of the RSW temperature field change, as shown in Figure 13 and Figure 14. From Figure 13, it can be observed that as the nickel layer thickness increases from 0.1 mm to 1.0 mm, the welding current density decreases from 395.2 A/mm^2^ to 284.5 A/mm^2^. According to the definition formula for current density, J = I/A, the increase in the nickel layer thickness allows for a more thorough current diffusion in the thickness direction, correspondingly increasing the effective conductive area and leading to a decrease in the current density. The electrical conductivity of nickel is higher than that of stainless steel. Based on the principle of current continuity and Ohm’s law, J = σE, an increase in the nickel layer thickness optimizes the current distribution path, enabling the current to pass through the nickel layer more uniformly and reducing the local current concentration. The increase in the nickel layer thickness also improves the interfacial contact state, diminishes the non-uniformity of the contact resistance, and promotes the expansion of the current distribution area. According to the Joule heat generation formula, changes in the current density directly affect the heat input. When the nickel layer thickness increases from 0.1 mm to 1.0 mm, the current density decreases by approximately 28%, correspondingly reducing the Joule heat generation rate, which is consistent with the decreasing trend of peak temperature discussed earlier. The temperature gradient in the heat conduction equation q = −k∇T is influenced by the current density distribution, and the high thermal conductivity of nickel accelerates heat diffusion, making the current density distribution more uniform. Comprehensive analysis indicates that a nickel layer thickness in the range of 0.3–0.5 mm yields an appropriate current density of 362.1–335.0 A/mm^2^. This range ensures a sufficient energy input for effective nugget formation while avoiding issues such as expulsion and electrode sticking caused by an excessively high current.

The peak temperatures on the different thicknesses of the nickel layer are shown in Figure 14. When the nickel layer increases from 0.1 mm to 1.0 mm, the reduction in the contact resistance leads to a decrease in heat generation of approximately 25–30%. This is because of the high thermal conductivity of the nickel layer on the welding thermal process. According to Fourier’s law, an increase in the nickel layer thickness significantly enhances the heat flux density in the thickness direction, causing heat from the welding heat source center to rapidly diffuse along the axial direction. Simultaneously, the nickel layer acts as an additional thermal resistance layer, with its thermal resistance increasing along with thickness, further inhibiting heat accumulation in the interfacial region. Furthermore, the contact resistance contributing to Joule heat decreases as the nickel layer thickens. This is because the high electrical conductivity of nickel optimizes the interfacial contact state. The heat capacity effect of the nickel layer, C = ρVc, intensifies with increasing volume, requiring more thermal energy to achieve the same temperature rise.

Consequently, in the thin nickel layer region, dominated by changes in the contact resistance, the temperature decrease rate is approximately 60 °C/0.1 mm. In the medium-thickness region, the heat conduction effect becomes prominent, and the decrease rate slows to about 40 °C/0.1 mm. In the thick nickel layer region, the thermal resistance reaches saturation, and the decrease rate further reduces to approximately 15 °C/0.1 mm. Additionally, the nickel layer influences the thermal effects of interfacial reactions by altering the Fe–Ni–Cr ternary diffusion kinetics. An appropriate thickness can promote metallurgical bonding without generating excessive brittle phases. Under the conditions of an 8 kN electrode pressure, a 10 kA welding current, and a 400 ms current application time, a nickel layer thickness of 0.3–0.5 mm achieves optimal interfacial bonding quality and temperature distribution uniformity while ensuring a sufficient peak temperature.

**Figure 15:**
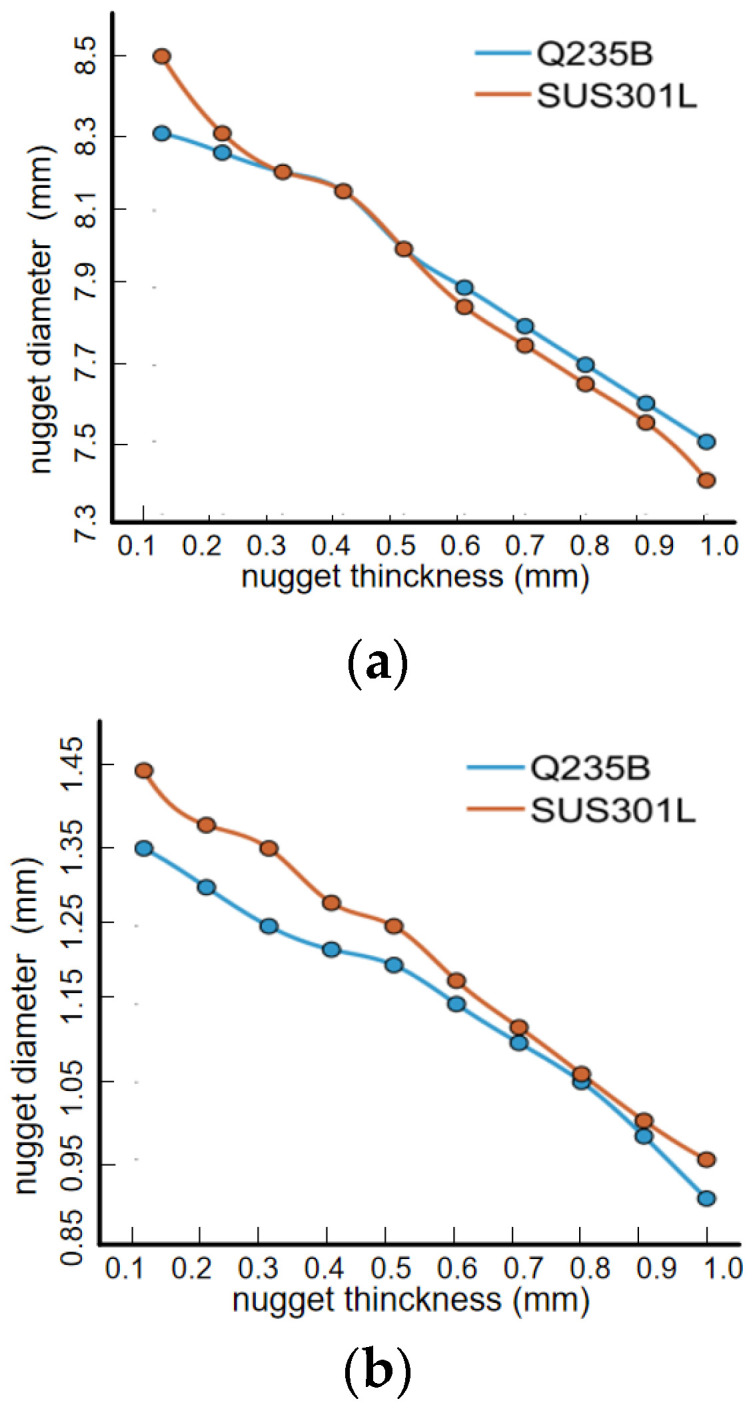
Effect of nickel layer thickness on nugget dimensions. (**a**) Effect of nickel layer thickness on nugget diameter. (**b**) Effect of nickel layer thickness on nugget height.

The effect of the nickel layer thickness on the nugget dimensions is shown in Figure 15. In the thin nickel layer region, the nickel layer fails to form a continuous and uniform interlayer, leading to differences in the thermal diffusion capabilities between the stainless steel side and the low-carbon steel side. According to the thermal diffusion equation, the lower thermal diffusivity on the stainless steel side results in more pronounced heat accumulation and more extensive nugget expansion, while the heat dissipates more rapidly on the low-carbon steel side, causing a significant difference in nugget dimensions between the two sides. As the nickel layer increases to 0.3–0.5 mm, it forms a complete thermal regulation layer. Its high thermal conductivity balances the heat distribution between the two materials, while the plastic deformability of the nickel layer promotes contact uniformity between the electrode and workpiece. Based on the contact resistance model, the increase in the nickel layer thickness enlarges the actual contact area, further reducing non-uniformity in interfacial resistance and minimizing the difference in the nugget dimensions between the two sides. When the nickel layer thickness exceeds 0.5 mm, the heat transfer reaches a quasi-steady state, with the thermal resistance of the nickel layer becoming the dominant factor. Heat dissipation and accumulation achieve a dynamic equilibrium, compressing the temperature field difference between the two sides to a minimum and keeping the nugget size difference relatively stable. Additionally, the heat capacity effect of the nickel layer intensifies with increasing volume, requiring more thermal energy to achieve the same temperature rise, which further contributes to the reduction in nugget dimensions.

## 5. Physical Experiment Comparative Analysis

### 5.1. Metallographic Analysis

**Figure 16:**
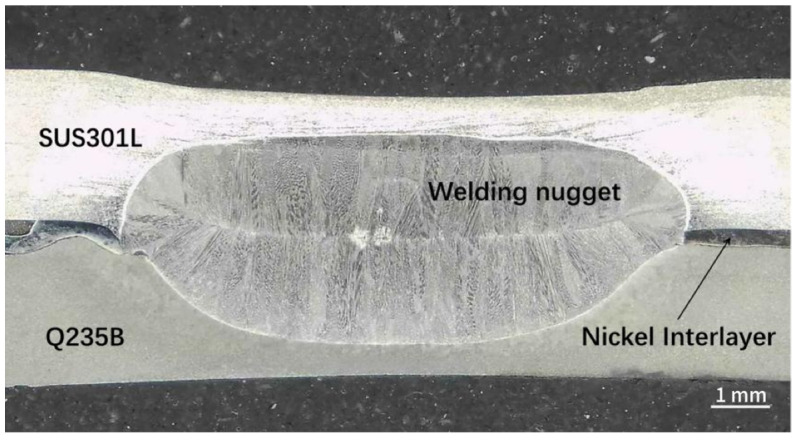
Macroscopic morphology of RSW joints.

Figure 16 shows the macroscopic morphology of the RSW joint. It can be clearly seen that after adding the nickel interlayer, the welding nugget is almost located at the center position of the stainless steel plate and the low-carbon steel plate, and the phenomenon of the melt core offset almost disappears. The melt depth and melt width can almost fully meet the requirements of engineering applications.

**Figure 17:**
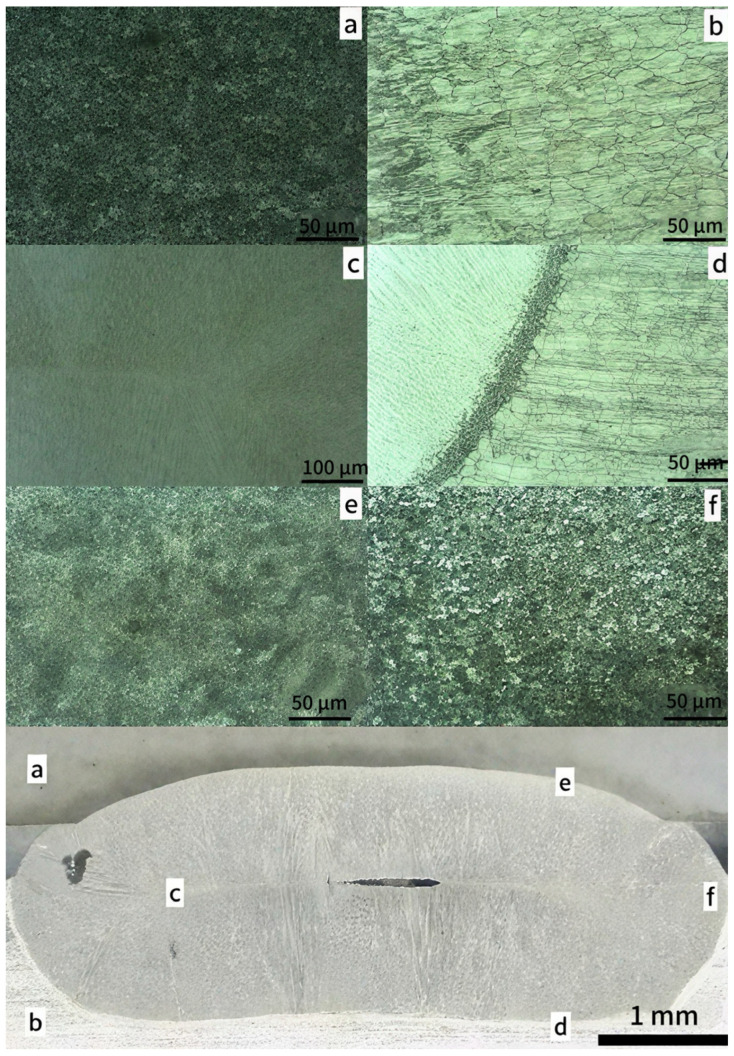
Metallographic structure graph of spot-welded joints. (**a**) Q235 matrix structure; (**b**) stainless steel matrix structure; (**c**) nugget zone structure; (**d**) structure of nugget and stainless steel heat-affected zone; (**e**) structure of nugget and low-carbon steel affected zone; (**f**) nickel layer heat-affected zone structure.

**Figure 18:**
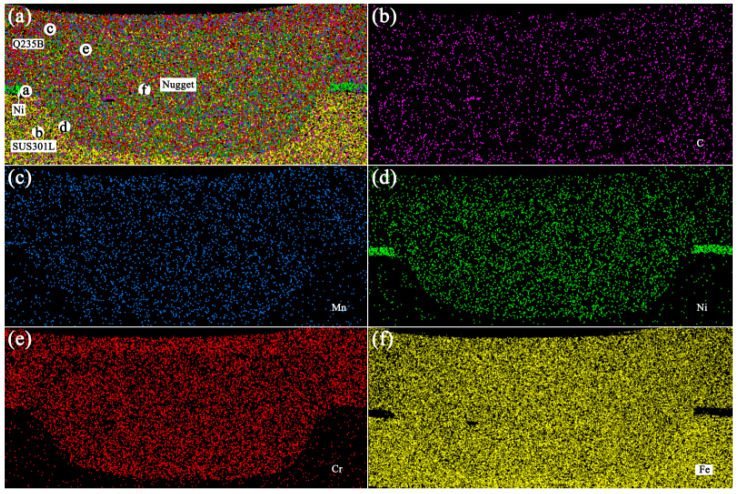
The common element distribution of spot welds. (**a**) All elements; (**b**) carbon; (**c**) manganese; (**d**) nickel; (**e**) chromium; (**f**) iron.

**Figure 19:**
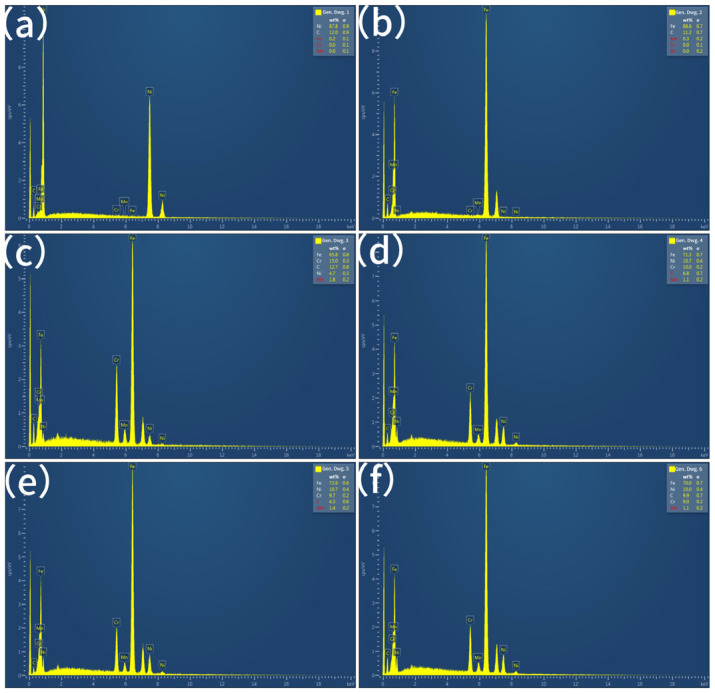
The elemental composition at each typical position in Figure 18a. (**a**) Ni interlayer; (**b**) Q235B base metal zone; (**c**) SUS301L base metal zone; (**d**) the HAZ (heat-affected zone,) on the SUS 301L side; (**e**) the HAZ on the Q235B side; (**f**) the nugget zone.

To further analyze the microstructural changes in each area of the RSW nugget, the metallographic morphologies of each area are shown in Figure 17. The distribution of several common elements such as Fe, C, Cr, Ni and Mn in the RSW joint was analyzed by scanning electron microscopy. The results are shown in Figure 18. The distributions of the common element contents in various typical areas of the welded joint were extracted, and the results are shown in Figure 19.

From the results of metallographic analysis and the energy spectrum analysis, it can be seen that the microstructure of the Q235 low-carbon steel base material is a two-phase structure of ferrite and pearlite. The ferrite is in an equiaxed crystal form, and the pearlite is in a lamellar arrangement. This area was less affected by the spot-welding thermal cycle, and the microstructure did not undergo significant changes. The energy dispersive spectrum (EDS) analysis confirmed that the Ni content in this area was zero, the Cr content was extremely low, the Fe and C elements were evenly distributed, and the Mn content was at a relatively low level.

The microstructure of the SUS301L base metal zone is a single-phase austenite structure, accompanied by an obvious annealing twinning, and the grains are equiaxed. Due to the poor thermal conductivity of stainless steel, the thermal influence in the base metal zone is slight, and no carbide precipitation or grain boundary sensitization occurs. The EDS shows that the Cr and Ni elements are significantly enriched, and the Mn content is higher than that of the Q235B. The point scan measurement indicates that the Ni content is approximately 4.7%, which is consistent with the composition characteristics of the SUS301L austenitic stainless steel.

The nugget is the product formed by the rapid solidification of the Q235B base metal, the SUS301L base metal and the intermediate nickel layer after they melt together. It forms an austenite–ferrite duplex structure. The EDS distribution results indicate that the Fe and C are uniformly distributed within the melt nucleus, while the Mn element is slightly higher on the stainless steel side than on the low-carbon steel side. The Ni and Cr elements are fully homogenized through the melting and recrystallization process, without any local segregation.

In Figure 19, the component analysis results from the six characteristic positions show that the nickel content in the middle nickel layer is 87.8%. After fully melting, it mixes with the base metal on both sides, resulting in a uniform and stable Ni content of approximately 10% within the weld nugget, which is much higher than the 4.7% of the SUS301 base metal. Moreover, there is no significant difference in the Ni content at the center of the weld nugget and at the edges on both sides. This indicates that during the melting and rapid solidification processes of the welding, the uniform distribution of the elements was effectively achieved. Nickel, as a strong austenite stabilizing element, not only inhibits excessive carbon diffusion and the formation of brittle carbides but also significantly reduces the tendency of martensitic transformation, making the melt nucleus mainly composed of an austenite–ferrite duplex structure with good toughness and significantly improving the toughness of the core area of the joint.

The organizational evolution and the element distribution in the interface transition zone exhibit a distinct gradient feature. There are local melting traces at the interface between the melt core and SUS301L, resulting in the formation of fine recrystallized grains. Although the peak temperature is close to the melting point, causing a slight growth of austenite grains, the diffusion of nickel inhibits the precipitation of carbides at the grain boundaries, avoiding grain boundary sensitization. Therefore, the microstructure in this area smoothly transitions from the melt core’s as-cast structure to the recrystallized structure of the stainless steel heat-affected zone, with a dense interface bonding. At the interface between the melt nucleus and the Q235B low-carbon steel under rapid cooling conditions, after the high-temperature austenitization, a martensitic transformation occurs, resulting in a coarse martensite and Widmanstätten structure. However, the diffusion of nickel elements significantly smooths the chemical composition gradient in this area, and the structure transforms into a mixed system of martensite, austenite, and a small amount of carbides, with continuous residual austenite films forming around the martensite lamellae. Therefore, it effectively alleviates the brittleness of the single martensite and enhances the interface bonding toughness.

From the above analysis, it can be seen that during the spot-welding process, the base materials on both sides are less affected by the thermal cycling, maintaining the original microstructure and composition characteristics. The addition of the intermediate nickel layer not only achieved the uniform distribution of the elements in the weld core through melting and recrystallization but also regulated the phase transformation behavior in the weld core and the interface area through the austenite stabilization effect, inhibiting the formation of brittle phases and grain boundary sensitization and simultaneously smoothing the chemical composition gradient at the interface. Finally, a continuous microstructure, a uniform composition, and a good interface bonding point weld joint was formed. This laid a microscopic foundation for the improvement of the joint’s mechanical properties.

### 5.2. Comparative of Simulation and Physical Experimental Results

**Figure 20:**
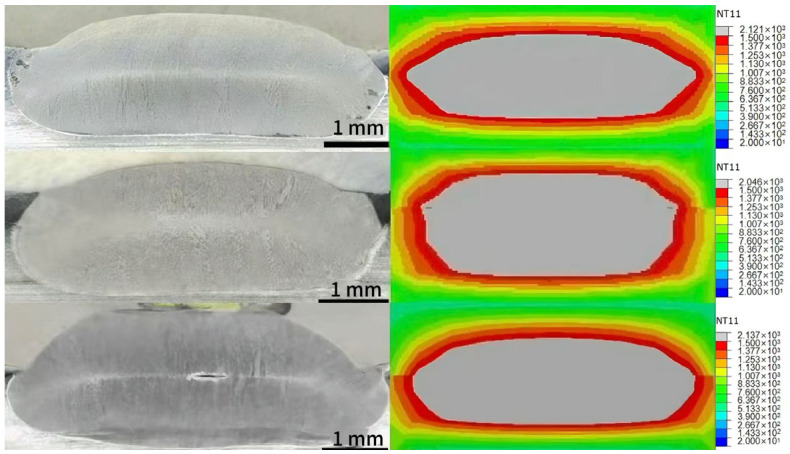
Results of the nuclear test and simulation results.

**Figure 21:**
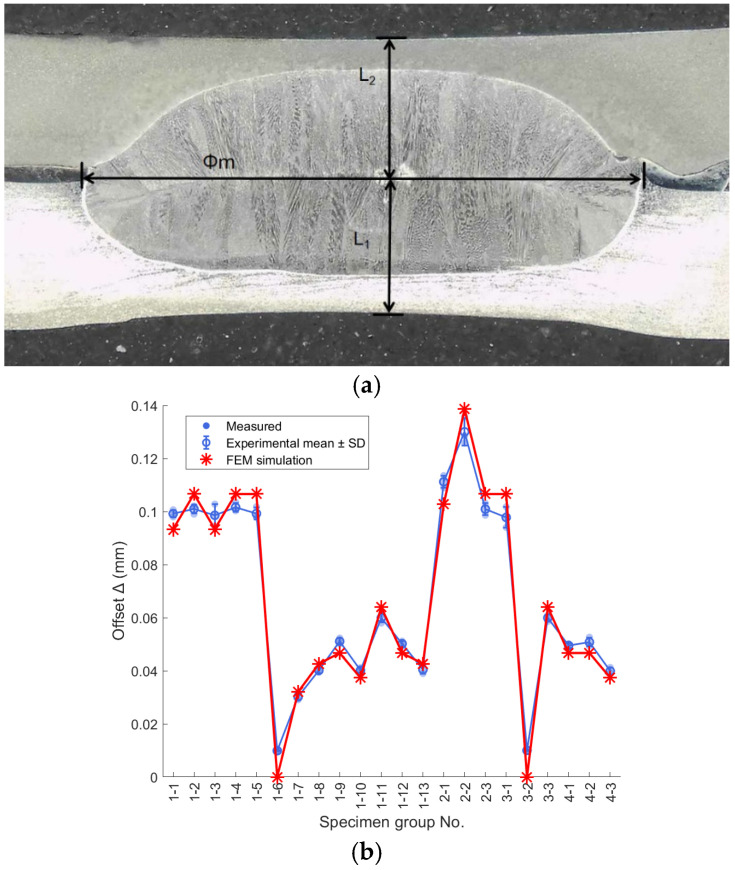

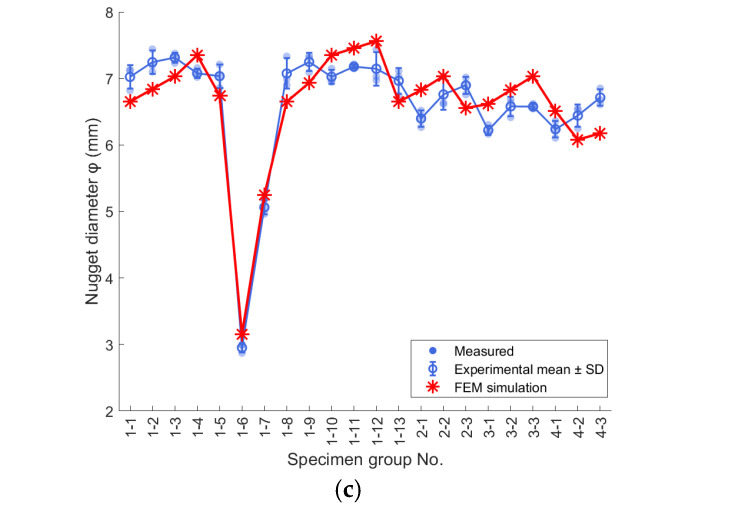
The comparison of FEA results and the metallographic measurements. (**a**) Schematic diagram of the measurement method for the size of the fusion core. (**b**) Measurement results of the melt core diameter. (**c**) Measurement results of the nuclear offset quantity.

The comparison between the finite element analysis results and the actual point weld joint morphology is shown in Figure 20. As can be seen from the Figure, the shape and size of the weld nugget are in good agreement with the results of the FEA (finite element analysis). Measure the distance from the center of the weld nugget to the upper surface of the SUS306L steel plate, which is recorded as L1. Measure the distance from the center of the weld nugget to the lower surface of the Q235B steel plate, which is recorded as L2. The weld nugget offset △ is the difference between L1 and L2. The diameters φ_f_ of the nugget in the FEA results and φ_m_ of the metallographic analysis were measured separately. The results are shown in Figure 21. From the quantitative comparison results, it can be seen that the error of the offset △ is approximately 6.6%, and the relative error of the joint diameter is about 5%, indicating a relatively good simulation accuracy.

There are several factors that may cause a relatively large error due to the offset of the weld core. Firstly, the uncertainty of the inherent material property parameters exists.

In actual production, there is a certain anisotropy between cold-rolled low-carbon steel plates and stainless steel plates. However, it is extremely difficult to obtain the complete anisotropic data of the materials under high-temperature conditions. Therefore, in this paper, the material properties are defined as isotropic materials. Thus, the dynamic changes of the thermal conductivity coefficient and the contact resistance under actual working conditions differ from the static parameters used in the simulation. Secondly, the influence of environmental factors during actual welding, including the surface condition of the workpiece, the distribution of the oxide layer, and the fluctuation of the environmental temperature, are variables that are difficult to completely reproduce in the model. Furthermore, the control accuracy of the equipment system, such as the current response characteristics and the pressure stability, will also introduce certain deviations in the statistics.

Although there are minor errors, the comprehensive analysis indicates that the actual geometric profile of the weld seam, the size of the weld core, and the morphology of the heat-affected zone are highly consistent with the simulation results. This suggests that the finite element model can effectively predict the evolution law of the temperature field and the formation mechanism of the weld core during the RSW process, providing a reliable theoretical basis and design guidance for the optimization of the process parameters.

### 5.3. Analysis of the Mechanical Properties

**Figure 22:**
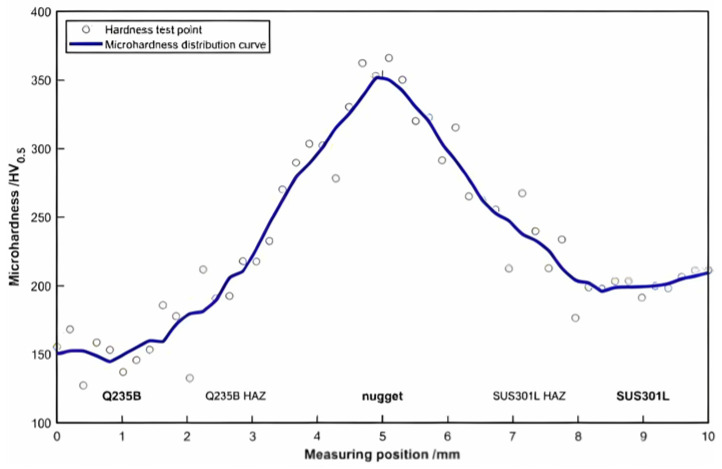
The microhardness distribution of the spot weld joint.

The microhardness distribution curve of the welding joint with a nickel interlayer is shown in Figure 22. The hardness was measured from the Q235B to the HAZ at Q235B side, to the nugget zone, to the HAZ at 301L side, and finally to the SUS301L.

The microhardness of Q235B is approximately 150–170 HV. After entering the heat-affected zone, the hardness rises rapidly, reaching approximately 300 HV. A peak in the microhardness occurs at the center of the fusion zone, reaching up to nearly 350 HV. Subsequently, the microhardness values gradually decrease towards the HAZ on the SUS301L side and finally stabilize at 180–220 HV in the base metal zone. This hardness distribution pattern is mainly attributed to the microstructure evolution caused by the welding thermal cycle and the diffusion behavior of the alloy elements. In the HAZ of the Q235B side, rapid heating and cooling during welding cause the austenite to transform into fine martensite and bainite, significantly enhancing the hardness. The fusion zone, as the intersection area of the different materials, experiences higher peak temperatures and faster cooling rates during the welding process. It is prone to form hardened structures such as high-carbon martensite and may also be accompanied by the migration and enrichment of the alloy elements (Cr, Ni, etc.), further increasing the hardness of this area. On the SUS301L side, due to its relatively high alloy content and austenitic stability, the hardening trend in the HAZ is relatively weak. The base material zone still maintains the typical austenitic structure, so the overall hardness is lower than that of the fusion zone. Compared with the dissimilar steel spot-weld joints without a nickel layer, the hardness distribution curve of this joint has better symmetry and continuity, providing a reliable metallurgical foundation for obtaining high-quality dissimilar steel weld joints.

**Figure 23:**
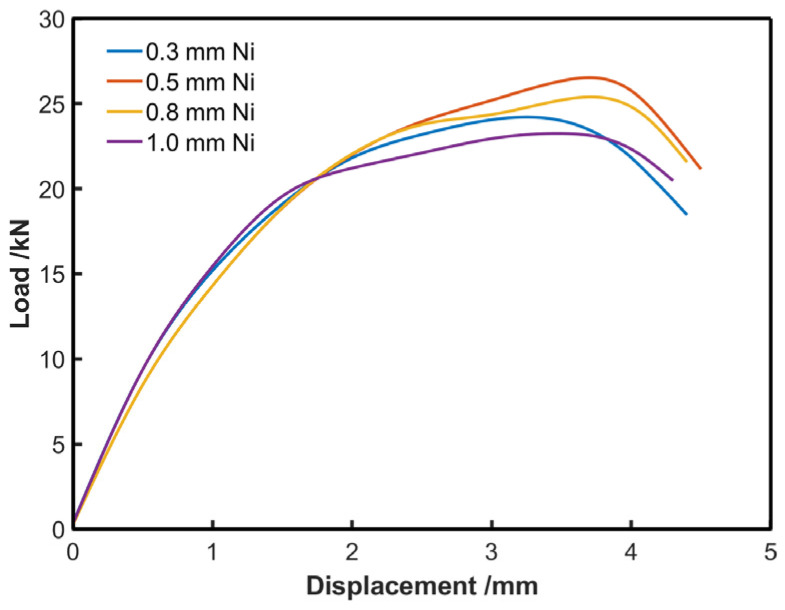
Stretching curves for different nickel layer thicknesses.

The tensile-displacement curves of the spot welds under the different nickel layer thicknesses are shown in Figure 23. The curve consists of the elastic stage, the plastic deformation stage, and the fracture stage. By comparing the curve characteristics, it can be seen that the joint with a 0.5 mm nickel layer has the highest peak load, the largest fracture displacement, the most gradual decline section, and the largest area under the curve. This indicates that it has the highest strength and the best toughness, and the fracture mode tends to be more similar to a ductile fracture feature. The peak load of the joint with a 0.8 mm nickel layer is approximately 25 kN, and the fracture displacement is about 4.4 mm. The comprehensive performance is slightly inferior to that of the joint with a 0.5 mm nickel layer. The peak load of the 0.3 mm nickel layer joint is approximately 24 kN, slightly lower than that of the 0.8 mm joint, while the fracture is similar. The joint peak load of the 1.0 mm nickel layer was the lowest, the fracture displacement was the shortest, the descending section was steeper, and the brittle characteristics were more obvious. The results show that if the thickness of the nickel layer is too thin, it may not be able to adequately buffer the physical performance differences between the different types of steel. If the thickness is too thick, it may lead to a performance degradation due to insufficient heat input or increased interface defects. An appropriate thickness of the nickel layer can not only effectively inhibit the excessive growth of brittle intermetallic compounds but also optimize the interface structure, thereby obtaining high-strength and high-toughness welded joints.

## 6. Conclusions

This study established a finite element model for the thermal–thermal multi-physics field coupling which was combined with an experimental verification to explore the influence of a nickel interlayer on the formation process of different material RSW joints. The main conclusions are as follows:

(1) The finite element analysis results show that the nickel interlayer effectively regulates the interface contact state and the current distribution. During the pre-pressing stage, although the nickel layer did not significantly affect the contact force between the electrode and the workpiece, it had a relatively obvious impact on the contact pressure distribution at the steel–nickel interface, significantly reducing the stress concentration and providing a conductive basis for the change in the current state during the energization stage. During the energization stage, the nickel layer acts as a resistance gradient transition layer, significantly reconstructing the current field in the welding zone, reducing the current concentration coefficient from 3.2 to below 1.5. This causes the current distribution to shift from being severely biased towards the low-carbon steel side to a more uniform and symmetrical pattern along the thickness direction, thereby improving the spatial distribution of the Joule heat source.
(2) The results of the physical experiments show that after the introduction of the nickel interlayer, the nickel element is uniformly distributed within the fusion nucleus, and the offset of the fusion nucleus is stably controlled within 0.15 mm. The joint melt nucleus is formed well, with ideal diameters, thicknesses, and symmetries. The microhardness along the interface shows a continuous and uniform transition. The mechanical performance analysis further confirms that selecting an appropriate thickness of the nickel interlayer can effectively improve the mechanical compatibility of the joint and significantly enhance its tensile strength.
(3) The simulation results of the finite element model and the actual morphology of the welding joint comparison results show that the error of the offset of the weld nugget simulation results is approximately 6.6%, while the error of the joint diameter is approximately 5%, indicating that the finite element analysis simulation accuracy is relatively good. This model provides a reliable theoretical tool for the in-depth understanding of the mechanism of the nickel interlayer’s effect and helps guide the development of RSW processes for different material steel plates.

## Figures and Tables

**Table 1:** Chemical composition (mass percentage) of plates.

Experimental Sheet	Mass Percentage							
	C	Si	Mn	P	S	Ni	Cr	N
SUS301L	0.024	0.37	1.12	0.026	0.002	7.53	17.71	0.11
Q235B	0.18	0.26	0.54	0.004	0.005	--	--	--
Ni	0.02	0.35	0.35	--	0.01	99	--	--

**Table 2:** Mechanical properties of plates.

Experimental Sheet	Mechanical Properties		
	Yield Stress (MPa)	Tensile Strength (MPa)	Strain Rate (%)
SUS301L	350	729	52.3
Q235B	235	450	24
Ni	390	450	45%

**Table 3:** Welding parameters.

Group No.	Nickel Thickness (mm)	Electrode Pressure (kN)	Welding Current (kA)	Current Time (ms)
1-1	0.3	8	8	400
1-2	0.3	8	9	400
1-3	0.3	8	10	400
1-4	0.3	9	8	400
1-5	0.3	9	9	400
1-6	0.3	9	10	150
1-7	0.3	9	10	200
1-8	0.3	9	10	300
1-9	0.3	9	10	400
1-10	0.3	10	8	400
1-11	0.3	10	9	400
1-12	0.3	10	10	400
1-13	0.3	11	10	400
2-1	0.5	8	8	400
2-2	0.5	8	9	400
2-3	0.5	8	10	400
2-1	0.8	8	8	400
2-2	0.8	8	9	400
2-3	0.8	8	10	400
2-1	1.0	8	8	400
2-2	1.0	8	9	400
2-3	1.0	8	10	400

## Data Availability

The original contributions presented in this study are included in the article. Further inquiries can be directed to the corresponding author.
